# Filtered and unfiltered lipoaspirates reveal novel molecular insights and therapeutic potential for osteoarthritis treatment: a preclinical *in vitro* study

**DOI:** 10.3389/fcell.2025.1534281

**Published:** 2025-02-27

**Authors:** Alissa Behn, Saskia Brendle, Marianne Ehrnsperger, Magdalena Zborilova, Thomas M. Grupp, Joachim Grifka, Nicole Schäfer, Susanne Grässel

**Affiliations:** ^1^ Department of Orthopaedic Surgery, Experimental Orthopaedics, Centre for Medical Biotechnology (ZMB/Biopark 1), University of Regensburg, Regensburg, Germany; ^2^ Research and Development, Aesculap AG, Tuttlingen, Germany; ^3^ Department of Orthopaedic and Trauma Surgery, Musculoskeletal University Center Munich (MUM), LMU Munich, Munich, Germany; ^4^ Department of Orthopedic Surgery, University of Regensburg, Asklepios, Germany; ^5^ Department of Trauma Surgery, University Medical Center Regensburg, Regensburg, Germany; ^6^ Department of Orthopedics and Ergonomics, Ostbayerische Technische Hochschule (OTH), Regensburg, Germany

**Keywords:** osteoarthritis, nanofat therapy, lipoaspirates, SVF, Adinizer^®^, Lipocube^TM^ Nano

## Abstract

**Introduction:**

Orthobiologics, such as autologous nanofat, are emerging as a potential treatment option for osteoarthritis (OA), a common degenerative joint causing pain and disability in the elderly. Nanofat, a minimally processed human fat graft rich in stromal vascular fraction (SVF) secretory factors, has shown promise in relieving pain. This study aimed to elucidate the molecular mechanisms underlying nanofat treatment of OA-affected cells and compare two filtration systems used for nanofat preparation.

**Methods:**

Chondrocytes and synoviocytes were isolated from articular cartilage and synovium of 22 OA-patients. Lipoaspirates from 13 OA-patients were emulsified using the Adinizer^®^ or Lipocube^™^ Nano filter systems to generate nanofat. The fluid phase of SVF from both filtered and unfiltered lipoaspirates was applied to OA-affected cells. Luminex multiplex ELISA were performed with lipoaspirates and cell supernatants alongside functional assays evaluating cell migration, proliferation, metabolic activity, and senescence.

**Results:**

A total of 62 cytokines, chemokines, growth factors, neuropeptides, matrix-degrading enzymes, and complement components were identified in lipoaspirates. Among these, significant concentration differences were observed for TIMP-2, TGF-ß_3_, and complement component C3 between the filtered and unfiltered samples. Nanofat enhanced chondrocyte proliferation and migration, as well as synoviocyte migration and metabolic activity, while reducing chondrocyte metabolic activity. Pain-related factors like β-NGF, MCP-1, Substance P, VEGF, and αCGRP were reduced, while anti-inflammatory TGF-β_1+3_ increased and pro-inflammatory cytokines (IL-5, IL-7, IL-15, and IFN-γ) decreased. Nanofat also elevated secretion of complement components and TIMPs in both cell types. Notably, our results revealed no significant differences in cellular effects between sSVF filtered using the Adinizer^®^ and Lipocube^™^ Nano systems, as well as compared to unfiltered sSVF.

**Discussion:**

Here, we provide first insights into how autologous nanofat therapy may ameliorate OA by enhancing chondrocyte proliferation and synoviocyte migration while modulating inflammatory and pain-related factors. However, further research is needed to determine its effects on cartilage regeneration.

## Graphical Abstract

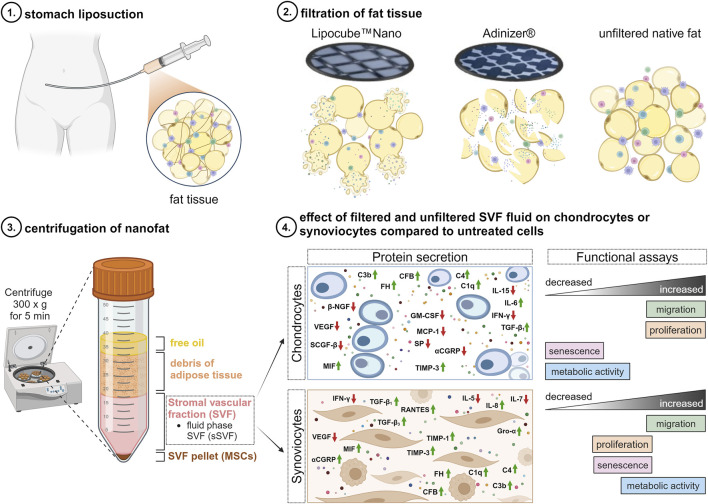

## Introduction

In 2020, Osteoarthritis (OA) affected over 500 million people worldwide, with hand, hip, and knee being the most impacted joints (Steinmetz et al., 2023). The pathogenesis of OA involves a complex interplay of mechanical forces, inflammation, and metabolic factors, affecting cartilage, subchondral bone, and synovium among other joint tissues (Hunter and Bierma-Zeinstra, 2019; Kulkarni et al., 2021).

Articular cartilage, composed of chondrocytes, is embedded in a vast extracellular matrix (ECM) including water, collagen, proteoglycans, and glycoproteins, and undergoes significant changes in OA. Chondrocytes in OA often display a senescent phenotype, differentiating into hypertrophic chondrocytes that contribute to cartilage degradation through the abnormal expression of matrix metalloproteinases (MMPs) and reduced collagen II synthesis (Goldring et al., 2011; Wang et al., 2004). Senescent chondrocytes display a senescence-associated secretory phenotype (SASP) with secretion of inflammatory factors that contribute to the joint’s pro-inflammatory environment. Similarly, senescent synovial fibroblasts, macrophages, and adipocytes exacerbate inflammation and joint damage (Childs et al., 2015).

Inflammed synovial tissue, recognized as a critical factor in OA pathophysiology, contains immune cells and cytokines that drive pain and structural damage (Benito et al., 2005). Studies have linked synovitis with pain, with recent findings showing that synovial inflammation correlates with pain sensation (Philpott et al., 2022). Although articular cartilage lacks blood vessels and nerve fibers, inflammatory processes can induce neoangiogenesis and nociceptive nerve fiber growth in joint tissues, leading to pain when cartilage is damaged (Ulici et al., 2024; Coaccioli et al., 2022).

Current OA treatments primarily focus on symptomatic relief through nonsteroidal anti-inflammatory drugs, acetaminophen, opioids, and intra-articular injections. However, these treatments often have limited efficacy and safety concerns. Disease-modifying OA drugs (DMOADs) aim to slow or reverse joint damage but face unresolved challenges due to disease heterogeneity (Hunter and Bierma-Zeinstra, 2019; Coaccioli et al., 2022; Schäfer and Grässel, 2022a).

Recent advances include the development of DMOADs targeting ECM homeostasis and chondrocyte metabolism. For example, the recombinant fibroblast growth factor 18, Sprifermin, promoted dose-dependent cartilage thickness but no significant changes in pain scores (Hochberg et al., 2019). An ADAMTS-5 inhibitor, S201086/GLPG 1972, and the WNT-β-catenin pathway inhibitor Lorecivivint (SM04690) have demonstrated potential in reducing cartilage loss and are currently under clinical investigation (Schäfer and Grässel, 2022a; Schnitzer et al., 2023; Kim et al., 2022).

Orthobiologics, such as autologous nanofat, introduced by Tonnard et al., in 2013, refers to mechanically emulsified lipoaspirates used in regenerative medicine, dermatology, and orthopedics. It has emerged as a promising new therapeutic option for alleviating pain in OA due to its regenerative potential and ability to modulate the inflammatory environment within joints. Its application in OA could offer a novel approach to managing the disorder, addressing not only pain relief but also potentially slowing disease progression (Tonnard et al., 2013). Unlike enzymatically prepared adipose-derived stromal vascular fraction, nanofat contains mesenchymal stem cells (MSCs), stromal cells, ECM macromolecules, and numerous paracrine factors (Trivisonno et al., 2019; Jeyaraman et al., 2021). Nanofat has shown comparable success to cellular stromal vascular fractions in reducing joint pain and improving mobility (Vargel et al., 2022).

This study aims to elucidate the molecular composition of a critical nanofat component, the fluid phase of the SVF (sSVF, containing both–intact adipocytes and secretomes of chopped adipocytes), on metabolism of chondrocytes and synoviocytes isolated from OA-affected knee joints. We compared nanofat prepared using the Lipocube™ Nano and Adinizer^®^ filter systems to each other and to unfiltered lipoaspirates. Soluble paracrine factors were analyzed with Luminex Multiplex-ELISA, and cellular responses were evaluated in OA-chondrocytes and OA-synoviocytes *in vitro*. This study aimed to deepen our understanding regarding the influence of nanofat on OA pain, progression and treatment outcomes by systematically identifying and analyzing its specific components and their effects.

## Methods

### Isolation and cultivation of human OA-chondrocytes and -synoviocytes

The experimental design of this study included human articular cartilage explants and synovial membranes prepared from knee joints of 22 OA-patients (Supplementary Table S1) after total knee replacement surgery. The use of human tissue was approved by the ethics committee at the University of Regensburg (ethics vote: 25-101-0189, ethikkommission@ur.de).

Chondrocytes were isolated as published previously (Köck et al., 2023). Synoviocytes (synovial fibroblasts and macrophages) were isolated by chopping the synovial membrane and digesting the pieces with Dispase II in PBS at 37°C for 2 h. Afterwards, the digested tissue was passed through a 70 μm cell strainer and cultured in DMEM/F12 with 10% FCS and 1% P/S. Both cell types were cultivated at 37°C, 5% CO_2_ and 95% humidity. For all further experiments, chondrocytes and synoviocytes at passage 1 and 2 were used.

### Preparation of lipoaspirates

Human adipose tissue was extracted from the abdomen of 13 OA-patients undergoing liposuction for nanofat pain therapy (Supplementary Table S1). The use of human tissue was conducted with full approval from the ethics committee at the University of Regensburg (ethics vote: 22-2915-101, Ethikkommission@ur.de). It is important to highlight that, in accordance with German regulations, the obtained nanofat emulsion must only be transplanted into sub-synovial fat tissue or the Hoffa’s fat pad. The proper placement of the application is monitored using sonography.

**FIGURE 1:**
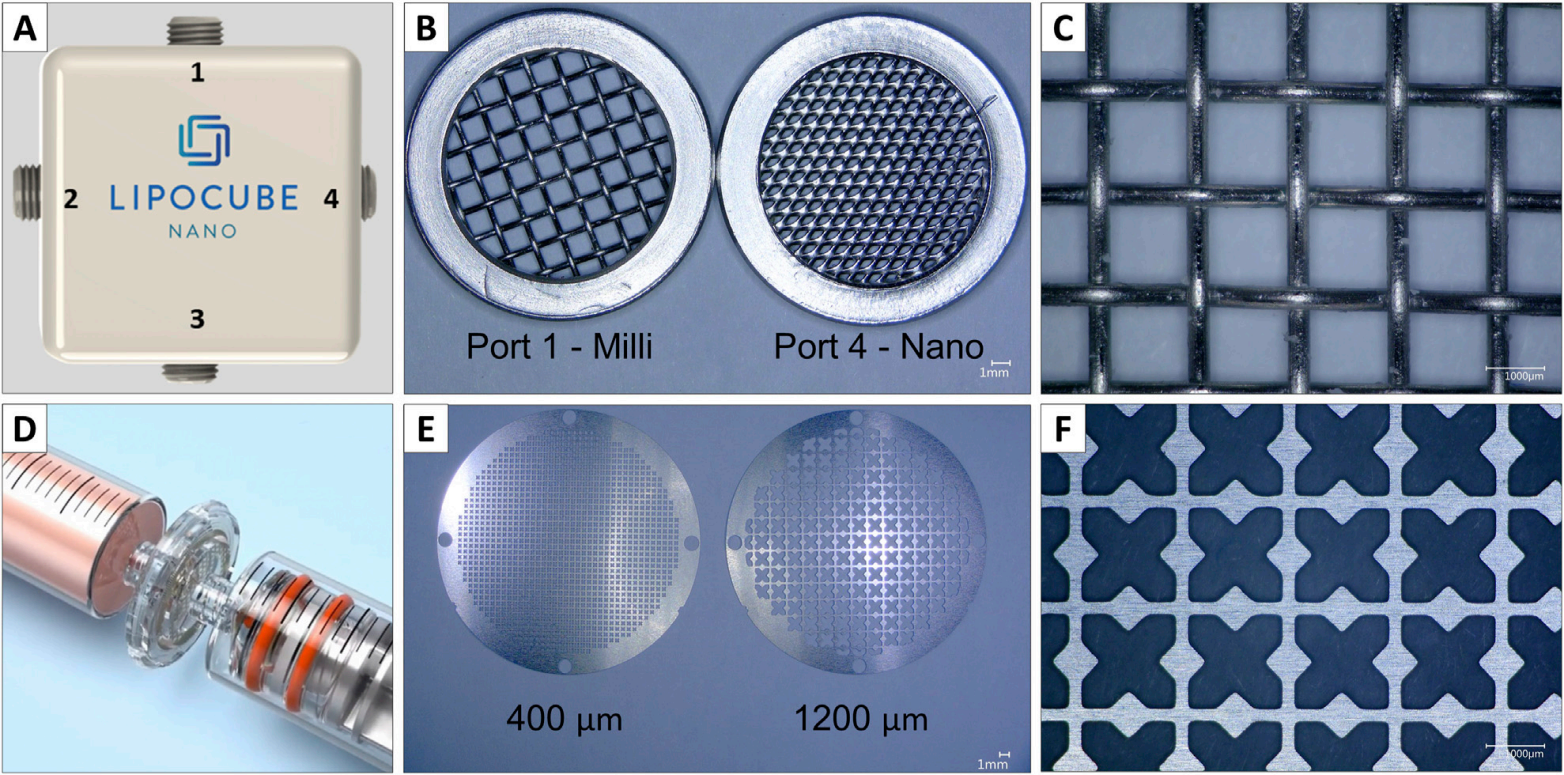
Comparison of Lipocube™ Nano filtration device and Adinizer^®^ Smart Kit. Two different filter devices were used to process lipoaspirates to nanofat **(A)** First, the Lipocube™ Nano device with four ports and **(B)** two integrated filters in port 1 (1,000 µm) and port 4 (500 µm) both with **(C)** blunt round blades **(D)** Second, the Adinizer^®^ filter system with two syringes and four individually attachable blades, **(E)** depicted two out of four filter discs, each of them with **(F)** double-edged, angular blades

Lipoaspirates were processed into nanofat using two different filter systems: the Lipocube™ Nano device (LC) (Lipocube, Inc., London, UK) (Cohen et al., 2019) and the Adinizer^®^ Smart Kit (AD) (BSLrest, South Korea). The Lipocube™ Nano is a device with a cuboid shape and four openings. Filtration started at the first opening with a 1,000 μm pore size filter blade. The fat tissue was compressed once from port 1 to port 2, then homogenized by passing 10 times from port 2 to port 3 (without filtration). Finally, microfat samples were compressed once from port 3 to port 4 through a 500 μm pore size filter blade (Figure 1A), resulting in LC-nanofat with a particle size of approximately 500 μm. Both integrated filter units contain blunt, round metal blades, which compress the lipoaspirate (Figures 1B, C).

In addition, the Adinizer^®^ Smart Kit with a different setup was used to process the fat tissue. Filtration started with a 2,400 μm pore size blade by connecting a syringe containing the lipoaspirate between the filter and another syringe (Figure 1D). The fat tissue was passed through the filter 5-10 times. Four filters were used sequentially (2,400, 1,200, 600, 400 μm). Unlike the blunt, round metal blades of the Lipocube, Adinizer filters feature double-edged, sharp metal blades. These allow for independent use and chopping the fat tissue rather than compressing it (Figures 1E, F). This process results in AD-nanofat with a particle size of approximately 400 μm.

**FIGURE 2:**
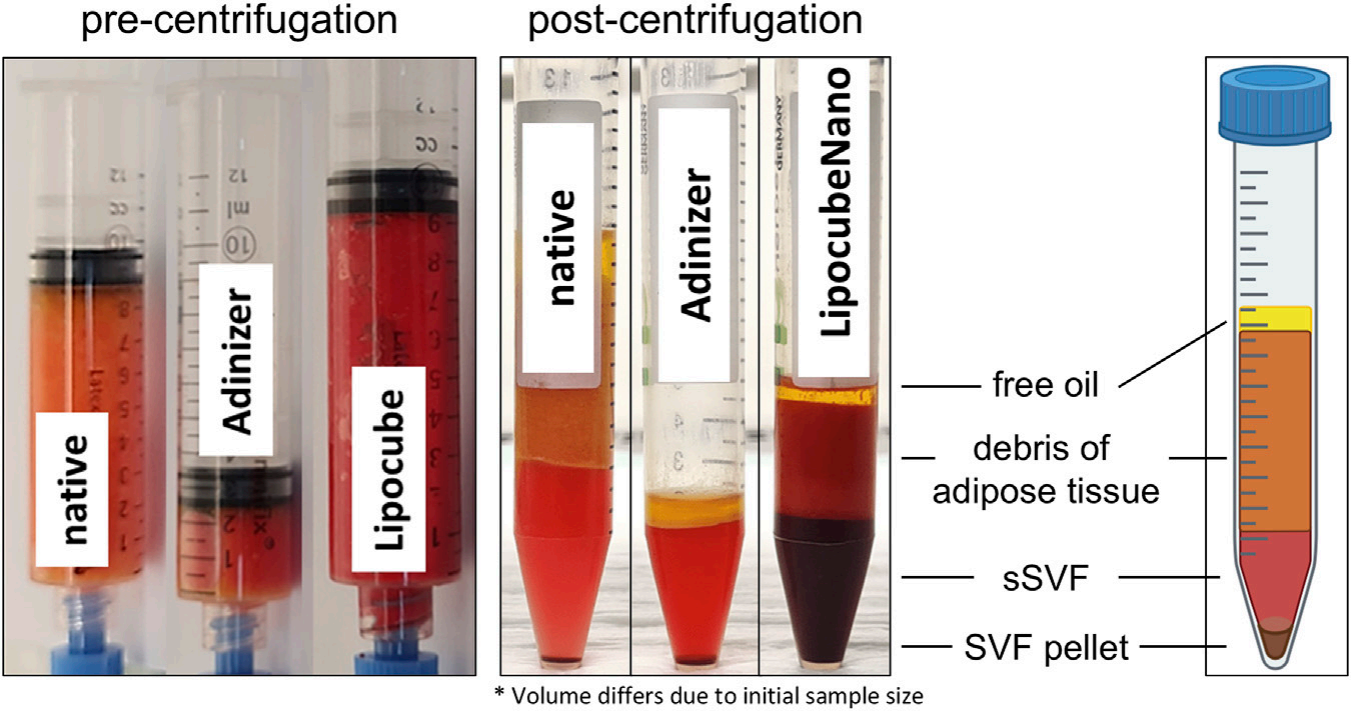
Processing of lipoaspirates. Native unfiltered lipoaspirates, Adinizer^®^-filtered nanofat and Lipocube™ Nano-filtered nanofat were centrifuged to obtain different fractions: free oil, debris of adipose tissue, fluid phase of SVF (sSVF) and the respective SVF pellet (containing MSCs). The fluid phase of the SVF (sSVF) was used for all further analyses, whereas the other fractions were disregarded.

As a control, lipoaspirates without filtration were used, further termed native lipoaspirate (NF-lipoaspirate). After filtration, LC-nanofat, AD-nanofat, and NF-lipoaspirate were centrifuged at 300 g for 5 min. The lipoaspirates were separated into different fractions: free oil, adipose tissue debris, the fluid phase of the stromal vascular fraction (sSVF, containing both–intact adipocytes and secretomes of chopped adipocytes), and the SVF pellet containing mesenchymal stem cells (MSCs) (from top to bottom, Figure 2). The fluid phase SVF (sSVF) was collected for further analysis, and stored at −80°C for long-term preservation. sSVF was used for further cell stimulation experiments at a dilution of 1:10, which was determined to be the most suitable concentration based on previous experiments.

### CellTiter-blue (CTB) viability assay

The metabolic activity of OA-chondrocytes and OA-synoviocytes was determined using the CellTiter-Blue (CTB) cell viability assay (#G8081, Promega GmbH.). Cells were seeded in DMEM/F12 containing 10% FCS and 1% P/S at a density of 20.000 cells/cm^2^. Then, OA-chondrocytes were treated with the sSVF of LC, AD and NF (dilution of 1:10) in chondrogenic medium (DMEM supplemented with 1% P/S, 110 μg/mL Sodium Pyruvate (#S8636; Sigma-Aldrich Chemie GmbH), 1 x ITS + premix Universal Culture Supplement (#354352; Corning Incorporated), 40 μg/ml L-Proline (#P5607; Sigma-Aldrich Chemie GmbH), 0.1 µM Dexamethasone (#D4902; Sigma-Aldrich Chemie GmbH), 10 ng/mL recombinant human TGF-ß3 (#100-36 E; Thermo Fisher Scientific Inc.), and 50 μg/mL Ascorbate-2-phosphate (A8960; Sigma-Aldrich Chemie GmbH)). OA-synoviocytes were incubated with LC-, AD-, NF-sSVF (1:10) in DMEM/F12 containing 5% FCS and 1% P/S. Following 24 h sSVF incubation, cells were treated with the CTB reagent and incubated for 2 h at 37°C. Fluorescence was measured at 545 nm excitation/590 nm emission.

### Cellular senescence

Senescence was assessed by measuring the activity of senescence-associated-β-galactosidase (SA-ß-gal) using the Cellular Senescence Assay kit (#CBA231, Cell Biolabs, Inc.), following the manufacturer’s protocol. Chondrocytes and synoviocytes were seeded in DMEM/F12 with 10% FCS and 1% P/S at a density of 20.000 cells/cm^2^, following incubation for 24 h with sSVF from LC, AD and NF (1:10) as previously described. Fluorescence was measured with 360 nm excitation and 465 nm emission.

### Cell proliferation

Cell proliferation was assessed using a BrdU ELISA kit (#11647229001; Hoffmann-La Roche Ltd.). Chondrocytes and synoviocytes, seeded at 20.000 cells/cm^2^, were cultivated in DMEM/F12 with 10% FCS and 1% P/S for 24 h. The medium was then replaced with sSVF (1:10) as described previously, and BrdU labeling solution was added for an additional 24 h. The labeled cells were fixed, incubated with anti-BrdU-peroxidase, and colorimetric changes were analyzed after adding the substrate solution, following the manufacturer’s protocol. Absorbance was measured at 450 nm.

### Cell migration

Migration (scratch) assay was performed using culture-inserts with two wells (#80209-150, ibidi GmbH) to provide improved reproducibility. OA-chondrocytes and synoviocytes were seeded at a density of 10.000 cells/well in DMEM/F12 with 10% FCS and 1% P/S for 24 h. After removing the inserts, the cells were treated with diluted sSVF (1:10) of NF, LC or AD in either chondrogenic medium (chondrocytes) or DMEM/F12 with 1% FCS and 1% P/S (synoviocytes). Migration of cells was determined by measuring gap closure over time, using 0 h as reference.

### Luminex multiplex-ELISA and CGRP-ELISA

OA-chondrocytes and OA-synoviocytes were cultivated and treated with sSVF as previously described. After 24 h of sSVF incubation, the cells were washed once with PBS. OA-chondrocytes were then cultured in chondrogenic medium, while OA-synoviocytes were maintained in DMEM/F12 medium with 1% FCS and 1% P/S for an additional 24 h. Following this incubation, supernatants were collected, analyzed, and stored at −80°C for long-term preservation. Luminex multiplex-ELISA of different proteins (Supplementary Table S2) was performed with cell supernatants (undiluted) and sSVF of LC, AD and NF (diluted 1:5, 50 µL) by using Bio-Plex 200 system with HTF (#171000205, Bio-Rad Laboratories).

The protein levels of calcitonin gene-related peptide (CGRP) in the sSVF and cell supernatants were analyzed using human CGRP-I EIA Kit (#EIA-CGRP-1, RayBiotech, Inc.). The assays were performed according to the manufactures’ protocol.

### Statistical analysis

Statistical analysis was performed using GraphPad Prism 10.2.3 software (GraphPad Software Inc.). Results are presented as boxplots (showing median and range from minimum to maximum) or tables (showing mean and standard deviation). The Kruskal–Wallis test with corrected Dunn’s *post hoc* test was applied. Comparisons were made between the sSVF of AD, LC and NF-treated groups compared to the untreated control group (w/o). A p-value of ≤0.05 was considered statistically significant.

## Results

This study aimed to identify pain- and inflammation-related factors present in the fluid phase of the stromal vascular fraction (sSVF) of lipoaspirates from OA-patients, which were mechanically emulsified using two distinct filter systems–the Adinizer^®^ and the Lipocube™ Nano system. The sSVF fraction contains both–intact adipocytes and secretomes of chopped adipocytes. Furthermore, we investigated the effects of sSVF treatment on the cell metabolism of OA-chondrocytes and -synoviocytes and compared the outcomes of the two filtration systems used.

### Novel detection of pain- and inflammation-related factors in nanofat-derived sSVF

To our knowledge, this is the first study to comprehensively analyze the inflammation- and pain-related factors within nanofat-derived stromal vascular fraction (sSVF) from OA patients, comparing filtered and unfiltered samples.

We identified differentially expressed inflammation- and pain-related cytokines, chemokines, and growth factors, as well as pain-associated neuropeptides and complement components, within the sSVF of lipoaspirates from OA patients. Concentrations did not significantly differ between Adinizer-filtered (AD)-, Lipocube Nano (LC)-filtered, and not-filtered (NF) sSVF (Supplementary Table S3).

Additionally, OA-associated matrix degradation factors, including matrix metalloproteinases (MMPs) and their inhibitors, tissue inhibitors of metalloproteinases (TIMPs), were detected. Five MMPs (MMP-1, -2, -3, -7, -9) were identified, however MMP-13 concentration was below detection limit. The highest concentrations included TIMPs (TIMP-1, TIMP-2, TIMP-3), suggesting a potential positive effect by ameliorating cartilage destruction. TIMP-2 concentration in LC-nanofat (35 pg/mL) was significantly increased compared to AD-nanofat (25 pg/mL) (Supplementary Table S4, marked green). Three isoforms of anti-inflammatory TGF-β (TGF-β_1 – 3_) could be detected, withTGF-β_3_ being significant lower in LC-nanofat (6 pg/mL) compared to AD-nanofat (9 pg/mL) (Supplementary Table S4, marked red). The concentrations of Calcitonin Gene-Related Peptide (CGRP) and Substance P (SP), the two sensory neuropeptides analyzed in NF-, AD- and LC-sSVF, were not significant different (Supplementary Table S4). Complement components from both the classical and alternative pathways were measured, with C3 concentrations being lowest in LC-nanofat (8 pg/mL) compared to AD-nanofat (42 pg/mL) and NF-sSVF (77 pg/mL) (Supplementary Table S4, highlighted in blue).

### Nanofat treatment induced chondrocyte proliferation and synoviocyte migration

**FIGURE 3:**
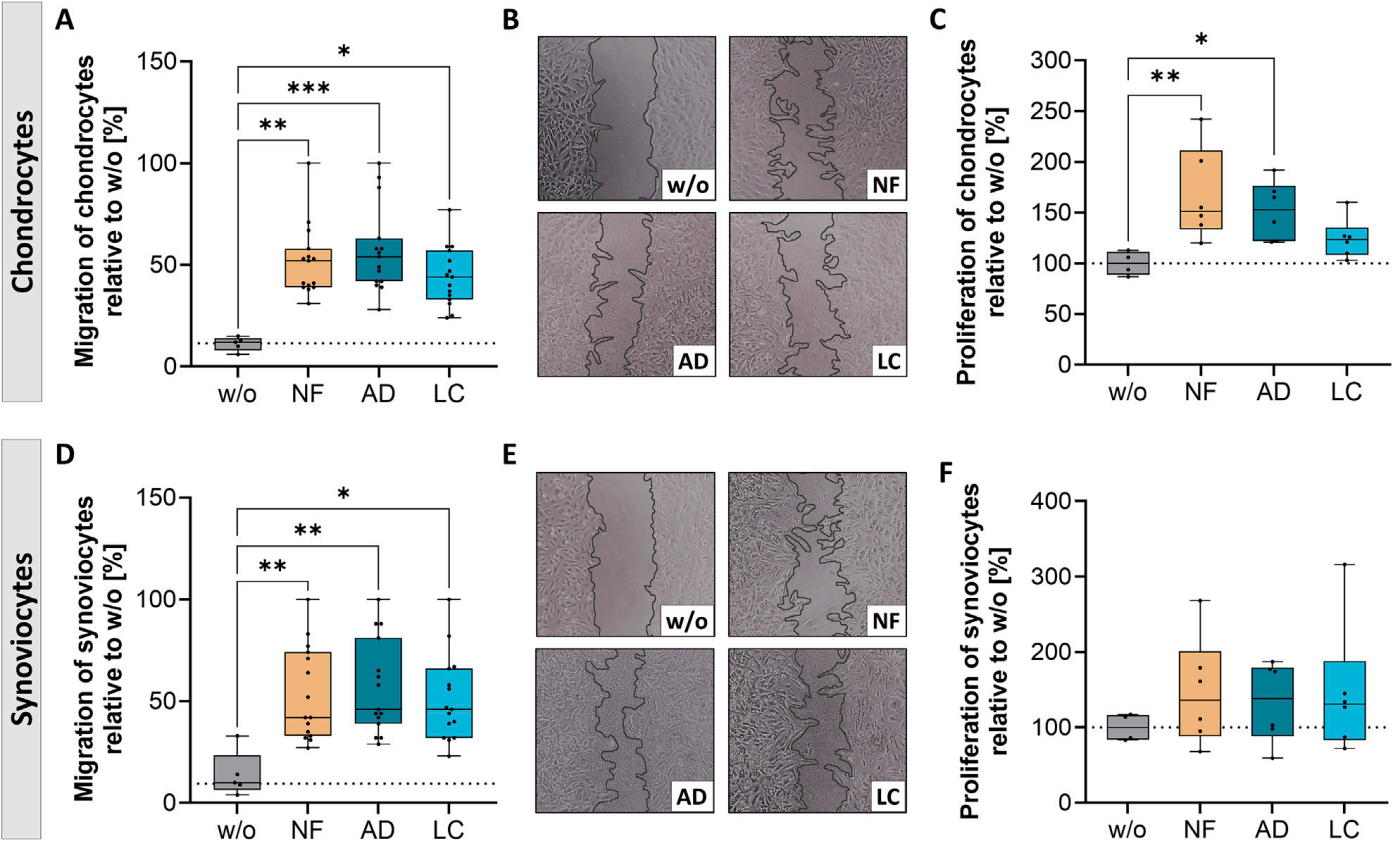
Nanofat treatment altered migration and proliferation of OA-chondrocytes and synoviocytes. Human OA-chondrocytes or synoviocytes were incubated for 24 h with either unfiltered sSVF (NF), Adinizer^®^-filtered (AD) or Lipocube™ Nano-filtered (LC) sSVF, or left untreated (w/o). Migration of **(A, B)** chondrocytes and **(D, E)** synoviocytes was increased after treatment with all three sSVF groups compared to w/o. Proliferation of **(C)** chondrocytes was induced after NF- and AD-sSVF incubation, whereas **(F)** synoviocytes proliferation was not impacted after sSVF treatment. n (chondrocytes) = 5; n (synoviocytes) = 5; n (NF, LC, AD) = 3. Kruskal–Wallis, Dunn’s multiple comparisons test, ***p < 0.001, **p < 0.01, *p < 0.05. (B, E) magnification ×10.

Considering the large range of factors identified in the sSVF and the established therapeutic benefits of autologous fat grafting in treating joint pain of OA-patients, we focused on investigating the metabolism of OA-chondrocytes and synoviocytes when exposed to LC-, AD- or NF-sSVF. We also compared the two different filtration systems used to assess whether there were significant differences in the metabolic cell data and to evaluate if both systems would be equally suitable for clinical application in joint treatments (Figure 3).

Migration of synoviocytes and chondrocytes was determined by measuring gap closure over time, using 0 h as reference (Figures 3A, B, D, E). Gap closure of chondrocytes was significantly enhanced after 24 h of AD- (mean 48% gap closure), LC- (mean 39.3% gap closure), and NF-sSVF (mean 47.5% gap closure) incubation compared to untreated controls (w/o, mean 11%) (Figures 3A, B). In addition, NF- and AD-sSVF significantly induced chondrocyte proliferation, whereas LC had no effect on proliferation (Figure 3C).

OA-synoviocytes migrated faster after 24 h of incubation with all three sSVF groups (mean NF 45.7%; AD 47.8%; LC 42.8%) compared to the untreated cells (mean 9.3%) (Figures 3D, E), whereas proliferation of OA-synoviocytes was unchanged (Figure 3F).

### Nanofat reduced metabolic activity of chondrocytes and synoviocytes

**FIGURE 4:**
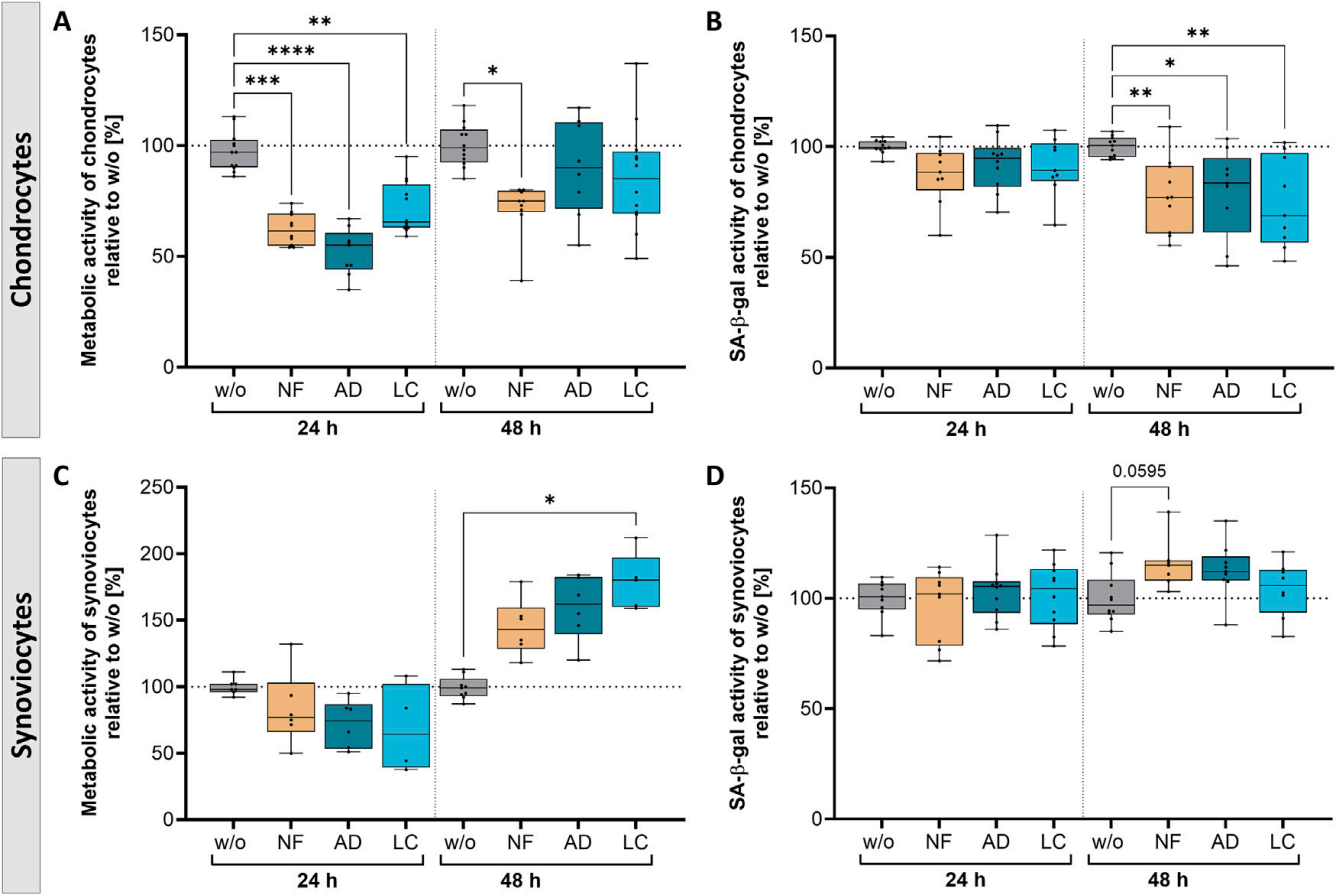
Nanofat treatment caused time-dependent alterations in the metabolic activity and senescence state of OA-chondrocytes and -synoviocytes. OA-chondrocytes or synoviocytes were incubated for 24 h and 48 h with either unfiltered sSVF (NF), Adinizer®-filtered (AD) or Lipocube^™^ Nano-filtered (LC) sSVF, or left untreated (w/o). Metabolic activity of **(A)** chondrocytes was time-dependently decreased, whereas **(C)** synoviocytes showed induced metabolic activity after 48 h of all three sSVF treatment groups compared to w/o. SA-β-gal activity as a marker for senescence induction was time-dependently reduced in **(B)** chondrocytes, but slightly increased in **(D)** synoviocytes 48 h after incubation with all three sSVF groups. n (chondrocytes) = 4; n (synoviocytes) = 4; n (NF, LC, AD) = 2–4. Kruskal–Wallis, Dunn’s multiple comparisons test, ****p < 0.0001 ***p < 0.001, **p < 0.01, *p < 0.05. SA-β-gal = senescence-associated ß-galactosidase.

To evaluate the therapeutic impact of nanofat treatment on cellular function and aging, the metabolic activity and senescent state of treated OA-chondrocytes and synoviocytes were determined (Figure 4).

Chondrocytes incubated for 24 h with NF-, AD- and LC-sSVF showed a significant reduction of metabolic activity compared to untreated control cells. This reduction shifted over time to match the levels observed in untreated cells when chondrocytes were incubated with AD- and LC-sSVF for 48 h (Figure 4A). The senescent state was correlated to senescence-associated ß-galactosidase (SA-β-gal) activity. Chondrocytes showed a time-dependent significant reduction in SA-β-gal activity when treated for 48 h with NF-, AD-, and LC-sSVF compared to untreated chondrocytes (Figure 4B).

The metabolic activity of synoviocytes was induced after 48 h of incubation with LC-sSVF and only by trend elevated in NF- and AD-sSVF-treated cells compared to the untreated controls (Figure 4C). No relevant alterations in SA-β-gal activity were observed when synoviocytes were incubated with the three sSVF groups, neither for 24 h nor for 48 h (Figure 4D).

We did not observe major differences in the metabolic effects on chondrocytes and synoviocytes between nanofat filtered with the Adinizer^®^ and Lipocube™ Nano filter systems.

### Nanofat suppressed secretion of pain-related factors of chondrocytes and synoviocytes

**FIGURE 5:**
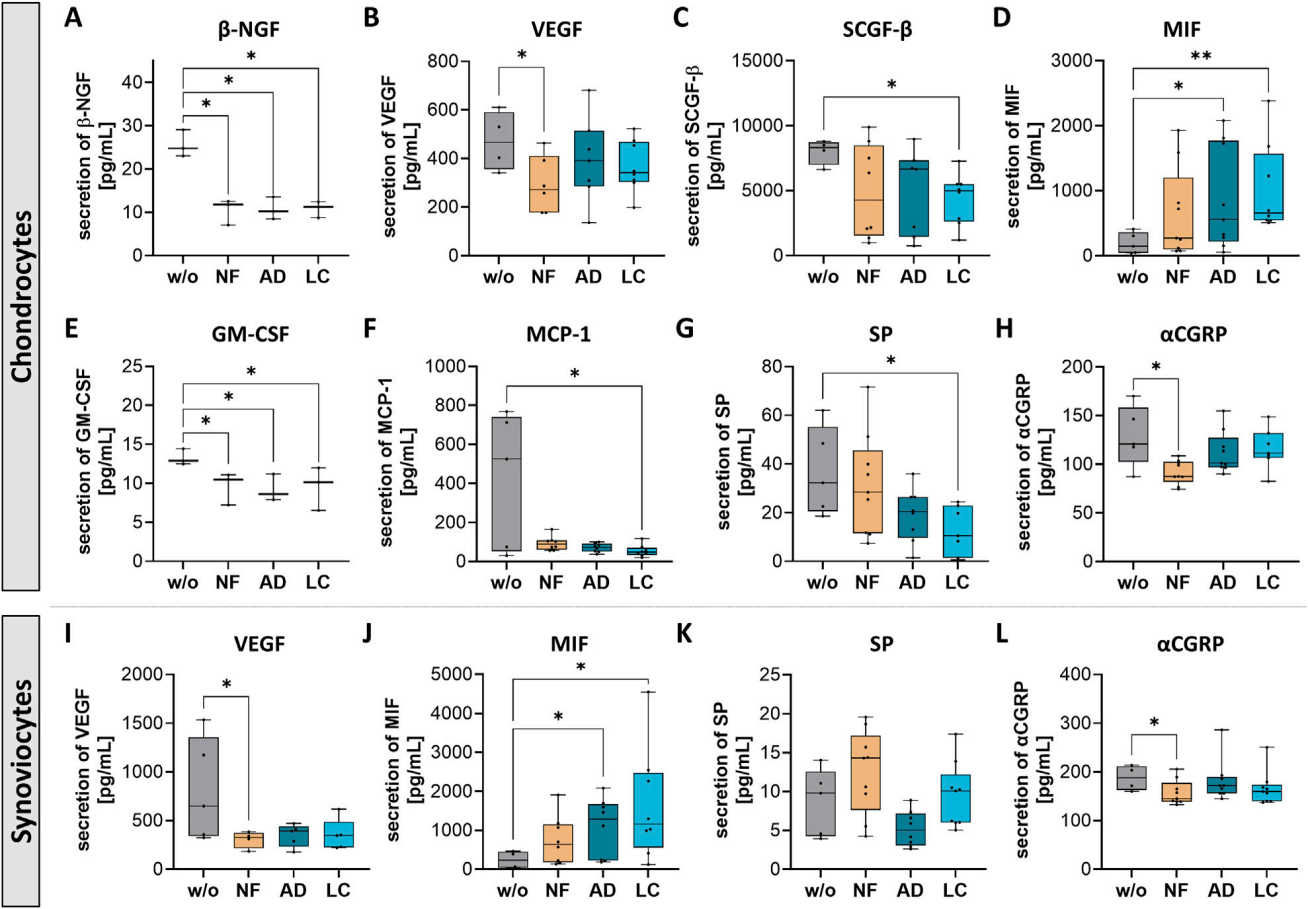
Nanofat treatment modified protein secretion levels of pain-related factors in chondrocytes and synoviocytes. Human OA-chondrocytes or synoviocytes were incubated for 24 h with either unfiltered sSVF (NF), Adinizer^®^-filtered (AD) or Lipocube™ Nano-filtered (LC) sSVF, or left untreated (w/o). Alterations of chondrocytes protein secretion levels could be detected for **(A)** β-NGF **(B)** VEGF **(C)** SCGF-β **(D)** MIF **(E)** GM-CSF **(F)** MCP-1 **(G)** Substance P (SP) and **(H)** α-CGRP. Secretion levels of sSVF-treated OA synovioctes changed for **(I)** VEGF **(J)** MIF **(K)** SP and **(L)** α-CGRP. n (chondrocytes) = 3; n (synoviocytes) = 3; n (NF, LC, AD) = 3. Kruskal–Wallis, Dunn’s multiple comparisons test, **p < 0.01, *p < 0.05.

Following the results showing that treatments with NF-, LC-, and AD-sSVF alter cellular behavior, the secretion profiles of chondrocytes and synoviocytes treated with these lipoaspirates were further analyzed. We performed Luminex Multiplex-ELISA to determine changes of pain- and inflammation-associated cytokines, chemokines and growth factors as well as pain-associated neuropeptides and complement components in the secretome (cell culture supernatants) of OA-chondrocytes and -synoviocytes (Figure 5).

Chondrocytes treated with NF-, AD- and LC-sSVF secreted less β-nerve growth factor (β-NGF), which is critically implicated in the sensitization and activation of nociceptors, the sensory nerves in joint tissues that respond to painful stimuli (Figure 5A). Vascular endothelial growth factor (VEGF), a promoter of angiogenesis and nerve growth, which also contributes to pain perception, was less secreted in chondrocytes (Figure 5B) and synoviocytes (Figure 5I) following NF-treatment compared to controls. Stem cell growth factor-beta (SCGF-β) secretion levels were reduced when chondrocytes were incubated with LC-sSVF (Figure 5C). Macrophage migration inhibitory factor (MIF) was significantly upregulated in secretomes of chondrocytes (Figure 5D) and synoviocytes (Figure 5J) treated with AD- and LC-sSVF. Additionally, chondrocyte secretion of granulocyte-macrophage colony-stimulating factor (GM-CSF) was significantly decreased when incubated with NF-, AD- or LC-sSVF (Figure 5E). Secretion of Monocyte chemoattractant protein-1 (MCP-1/CCL2), a critical mediator of inflammation and tissue remodeling in OA, was significantly reduced when cells were incubated with LC-sSVF (Figure 5F). Substance P (SP), which is associated with chronic inflammation and nociceptive pain in the joint, was slightly decreased in OA-chondrocyte (Figure 5G) as well as in synoviocyte secretomes (Figure 5K) when treated with LC- an AD.

Calcitonin gene-related peptide (αCGRP), a sensory neuropeptide involved in both pro-inflammatory and bone-protective properties, was decreased in the secretomes of chondrocytes (Figure 5H) and synoviocytes (Figure 5L) following treatment with NF-sSVF compared to untreated controls.

We did not observe any differences in the affected pain sensitization factors between nanofat filtered with the Adinizer^®^ and Lipocube™ Nano filter systems.

### Nanofat treatment modulated secretion of inflammatory factors in chondrocytes and synoviocytes

**FIGURE 6:**
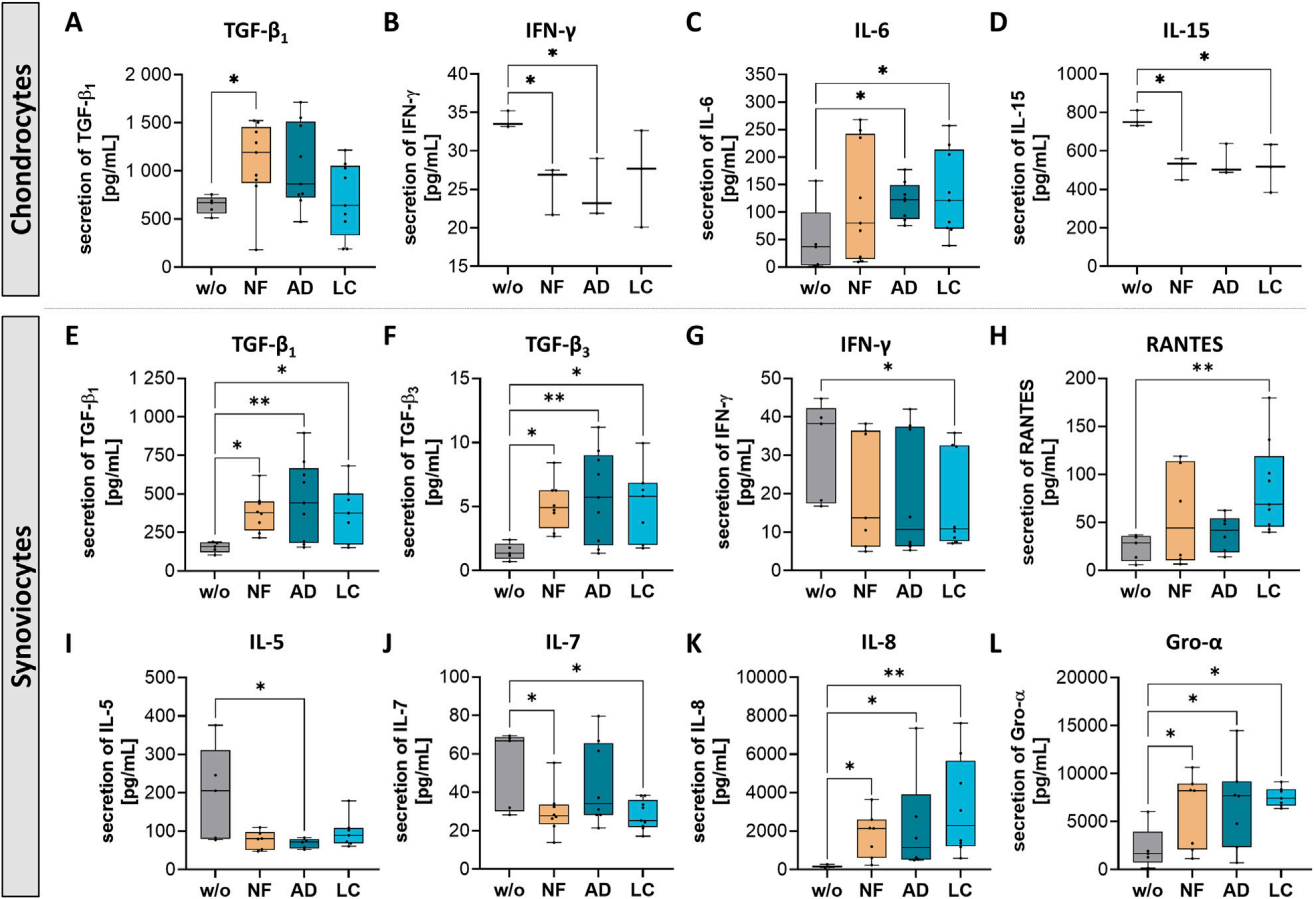
Nanofat treatment altered the protein secretion levels of inflammation-associated factors in chondrocytes and synoviocytes. Human OA-chondrocytes or synoviocytes were incubated for 24 h with either unfiltered sSVF (NF), Adinizer^®^-filtered (AD) or Lipocube™ Nano-filtered (LC) sSVF, or left untreated (w/o). Secretion levels of all three sSVF groups-treated chondrocytes were changed for the following inflammation-associated factors **(A)** TGF-β_1_, **(B)** IFN-γ **(C)** IL-6 and **(D)** IL-15. Synoviocytes secretion of **(E)** TGF-β_1_ **(F)** TGF-β_3_, **(G)** IFN-γ **(H)** RANTES, **(I)** IL-5 **(J)** IL-7, **(K)** IL-8 and **(L)** Gro-α was impacted by all three sSVF treatment groups. n (chondrocytes) = 3; n (synoviocytes) = 3; n (NF, LC, AD) = 3. Kruskal–Wallis, Dunn’s multiple comparisons test, **p < 0.01, *p < 0.05.

Secretion changes of transforming growth factor beta (TGF-β) and inflammation-related factors as interleukins (IL-5, 6, 7, 8, 15), interferon γ (IFN-γ), chemokine ligand 5 (CCL5; also known as RANTES) and chemokine ligand 1 (CXCL1; also known as Gro-α) were observed in with all three sSVF-treated chondrocytes and synoviocytes (Figure 6).

A significant increase in TGF-β_1_ secretion was observed in chondrocytes incubated with NF-sSVF compared to untreated control chondrocytes (Figure 6A), whereas synoviocytes treated with NF-, AD- and LC-sSVF secreted higher levels of TGF-β_1_ and TGF-β_3_ compared to untreated controls (Figures 6E, F).

Secretion of Interferon gamma (IFN-γ), considered as pro-inflammatory cytokine, was significantly reduced in NF- and AD-sSVF treated chondrocytes (Figure 6B) and in LC-sSVF treated synoviocytes (Figure 6G). For IL-15, a similar effect could be detected, with decreased secretion levels in the supernatant of NF- and LC-sSVF treated chondrocytes (Figure 6D). In contrast to that, pro-inflammatory cytokine IL-6 was elevated in secretomes of chondrocytes incubated with AD- and LC-sSVF (Figure 6C).

OA-synoviocytes are predominantly located in the synovium, which is recognized as the site of inflammatory processes (synovitis) in OA. In OA-synoviocytes, NF-, LC- and AD-sSVF treatment significantly reduced pro-inflammatory factors IL-5 (Figure 6I) and IL-7 (Figure 6J) compared to untreated cells. Additionally, secretion levels of IL-8 (Figure 6K), RANTES (Figure 6H) and Gro-α (Figure 6L) were elevated by synoviocytes following treatment with NF-, AD- and LC- sSVF.

### Nanofat impacted the secretion of complement components in chondrocytes and synoviocytes

**FIGURE 7:**
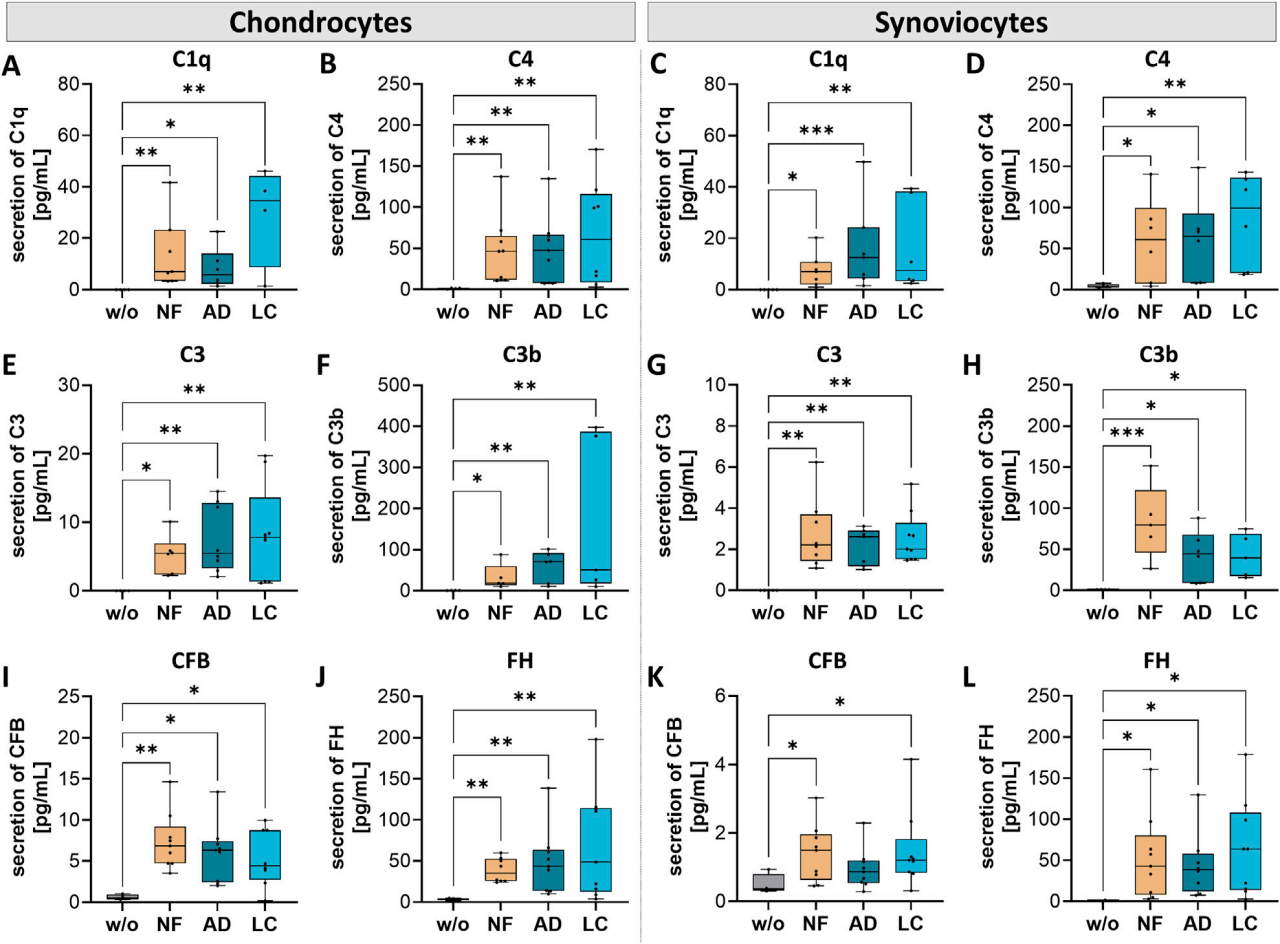
Nanofat treatment changed the protein secretion levels of complement components in chondrocytes and synoviocytes. Human OA-chondrocytes or synoviocytes were incubated for 24 h with either unfiltered sSVF (NF), Adinizer^®^-filtered (AD) or Lipocube™ Nano-filtered (LC) sSVF, or left untreated (w/o). Chondrocyte and synoviocyte secretion of complement components of the classical pathway **(A, C)** C1q **(B, D)** C4, and of the alternative pathway **(E, G)** C3 **(F, H)** C3b **(I, K)** CFB, and **(J, L)** FH was changed after incubation with all three sSVF-groups. n (chondrocytes) = 3; n (synoviocytes) = 3; n (NF, LC, AD) = 3. Kruskal–Wallis, Dunn’s multiple comparisons test, ***p < 0.001, **p < 0.01, *p < 0.05.

The complement system, essential to the innate immune system, serves as a primary defense mechanism and is involved in various physiological processes both systemically but also locally within nearly all cells of the body. We analyzed the secretion of complement components of OA-chondrocytes and OA-synoviocytes after incubation with nanofat (Figure 7).

Pre-incubation of NF-, LC- or AD-sSVF resulted in significant higher levels of C1q and C4 levels in chondrocyte (Figures 7A, B) and synoviocyte (Figures 7C, D) cell culture supernatants compared to untreated cells.

In both cell-types–chondrocytes and synoviocytes–NF-, AD- and LC-sSVF treatment leads to significantly elevated secretion of C3 (Figures 7E, G) and C3b (Figures 7F, H), the major complement component of the alternative pathway.

A significant elevation of complement factor B (CFB) secretion, crucial for the activation of the alternative pathway, was observed in treated chondrocytes (NF-, LC- and AD-sSVF) and synoviocytes (NF- and LC-sSVF) (Figures 7I, J). FH secretion, the main inhibitor of the alternative complement pathway, was significantly increased in OA-chondrocytes and synoviocytes treated with NF-, AD- and LC-sSVF compared to untreated cells (Figures 7J, L).

We did not observe any differences in the affected complement components between nanofat filtered with the Adinizer^®^ and Lipocube™ Nano filter systems.

### Nanofat impacted the secretion of tissue inhibitor of metalloproteinases (TIMPs) in chondrocytes and synoviocytes

Tissue inhibitors of metalloproteinases (TIMPs) regulate MMPs activity, which is crucial for maintaining cartilage integrity. In OA, an imbalance between MMPs and TIMPs can lead to excessive cartilage matrix degradation, contributing to disease progression.

**FIGURE 8:**
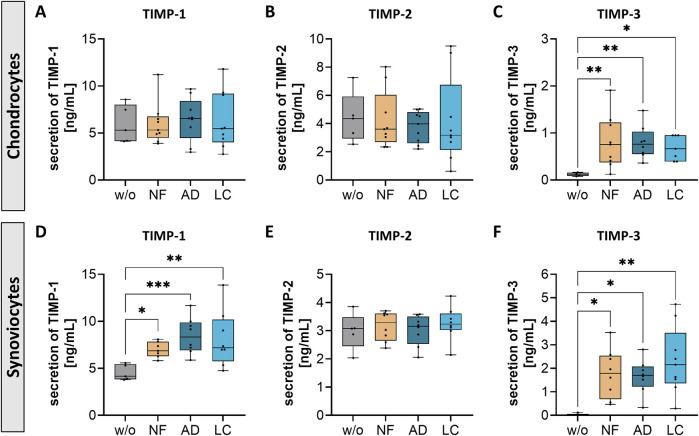
Nanofat treatment changed the protein secretion levels of TIMPs in chondrocytes and synoviocytes. Human OA-chondrocytes or synoviocytes were incubated for 24 h with either unfiltered sSVF (NF), Adinizer^®^-filtered (AD) or Lipocube™ Nano-filtered (LC) sSVF, or left untreated (w/o). Chondrocyte and synoviocyte secretion of **(A, D)** TIMP-1 and **(C, F)** TIMP-3 were changed after incubation with all three sSVF-groups **(B, E)** TIMP-2 secretion levels were not changed after sSVF incubation. n (chondrocytes) = 3; n (synoviocytes) = 3; n (NF, LC, AD) = 3. Kruskal–Wallis, Dunn’s multiple comparisons test, ***p < 0.001, **p < 0.01, *p < 0.05.

In this study, nanofat influenced TIMP secretion in both chondrocytes and synoviocytes. Treatment with NF-, AD-, and LC-sSVF led to elevated TIMP-3 levels in chondrocytes (Figure 8C) and increased TIMP-1 and TIMP-3 levels in synoviocytes (Figures 8D, F). However, the secretion levels of TIMP-1 remained unchanged in treated chondrocytes (Figure 8A), and no alterations were observed in TIMP-2 secretion in both chondrocytes and synoviocytes following treatment (Figures 8B, E). This modulation of TIMP secretion indicates that nanofat may help restore the balance between matrix degradation and repair processes in OA-affected joints.

## Discussion

The objective of this study was to elucidate the potential influence of nanofat on the progression and treatment outcomes of osteoarthritis (OA). This research aimed to provide first insights into the molecular mechanisms underlying autologous nanofat therapy for OA pain through *in vitro* analysis. The study conducted novel comprehensive analysis of pain-related and inflammation-associated factors present in unfiltered native lipoaspirates, and lipoaspirates processed using and comparing the Adinizer^®^ filter system and the Lipocube™ Nano filter system, with respect to effects on the molecular composition of the fluid phase stromal vascular fractions (sSVF) of the nanofat by the two different filter systems. Additionally, metabolic alterations in OA-chondrocytes and OA-synoviocytes treated with nanofat were examined.

The two emulsification devices, Adinizer^®^ and Lipocube™ Nano filters, are configured differently. A key distinction lies in the filter pore size; the Adinizer^®^ system, with a smaller pore size of 400 μm, segments the adipose tissue into finer conglomerates with sharp blades compared to the Lipocube™ Nano, which has a pore size of 500 µm with rounded blades. Kharamatsova et al. demonstrated that reducing the pore size of filters resulted in a different shape of adipocyte conglomerates (Khramtsova et al., 2020). Also, the number of viable adipocytes is significantly lower in Adinizer-processed lipoaspirates compared to Lipocube Nano-processed nanofat. The typical diameter of adipocytes is 80 μm, but this size can expand to 120 μm in obese individuals (Li and Spalding, 2022).

We hypothesized that the smallest pore size of the Adinizer filter of 400 μm and the sharp blades more effectively fragment adipose tissue and chop adipocytes compared to the Lipocube Nano, where adipocytes are more likely to remain intact due to less frequent compression and a larger rounded pore size. The Adinizer facilitates multiple compressions, enhancing adipocyte destruction, unlike the single compression through the Lipocube Nano.

In this study, we used the sSVF, hypothesizing that the soluble factors within the SVF containing both intact adipocytes and secretomes of chopped adipocytes, rather than the SVF pellet containing the mesenchymal stem cells (MSCs), are responsible for achieving beneficial effects in pain therapy for OA-patients. Notably, our findings showed no significant differences in cellular effects between sSVF filtered with the Adinizer^®^ and Lipocube™ Nano systems. This suggests that the different filter systems may have no significant impact on how adipocytes are processed, at least within our experimental setup. Some variations were observed regarding the composition of pain- and inflammation-related markers, specifically TIMP-2, TGF-β3, and complement component C3, between the Adinizer^®^- and Lipocube™-filtered sSVF. Notably, most of the clinical studies have documented a long lasting significant improvement of pain perception after application of processed/filtered autologous lipoaspirates into OA-affected knees making this rather simple and safe treatment technique attractive for delaying final joint replacement surgery. However, one should not ignore the possibility of inter-human variability which is fundamental for the success of these biological therapies, because a particular subgroup of patients could respond better/worse to a specific biologic stimulus in the sSVF than another.

Nanofat, containing metabolically active factors and MSCs, is recognized for its regenerative potential, impacting on surrounding tissues and cells (Grünherz et al., 2019). Here, we detected 62 cytokines, chemokines, growth factors, neuropeptides and factors associated with tissue degradation (MMPs and TIMPs) as well as complement components in the sSVF. All these factors have potential effects on cells within the joint. Therefore, sSVF-treated OA-chondrocytes and -synoviocytes were functionally analyzed regarding metabolic changes. OA-chondrocytes exhibited accelerated gap closure, which appears to result from both enhanced proliferation and increased migratory capacity. Numerous *in vitro* studies have indicated that chondrocytes can migrate under the influence of various factors (Morales, 2007). Moreover, migration of chondrocytes to an injury site and evidence of extracellular matrix (ECM) synthesis have been observed in *vitro* and *ex vivo* studies (Seol et al., 2014; Lyman et al., 2012). Notably, cartilage ECM reconstruction requires chondrocytes to be present at injury sites. An increase in chondrocyte proliferation boosts their numbers within the cartilage, which may be crucial for supporting effective cartilage regeneration (Le et al., 2020).

Contrary, synoviocytes showed enhanced migratory capacity with unaltered proliferation after sSVF treatment. Synoviocytes comprise a heterogeneous population of cells within the joint synovium, including fibroblast-like and macrophage-like cells. It is well established that macrophages and monocytes are motile, enabling them to reach any inflamed tissue in the body (Coaccioli et al., 2022). Macrophages can differentiate into the pro-inflammatory (M1) or anti-inflammatory (M2) phenotype. In synovial tissue, M1 as well as M2 macrophages are present, latter secreting anti-inflammatory cytokines and contribute to resolution of inflammation and regeneration of tissue defects (Mantovani et al., 2013; Zhao et al., 2023). Based on our current findings, we hypothesize that nanofat may modulate macrophage polarization via its soluble constituents, potentially enhancing M2 macrophage migration and positively affecting the joint’s inflammatory milieu. In addition, metabolic activity of lipoaspirate-treated OA-chondrocytes was found to be significantly reduced, and the senescent state was decreased in NF-lipoaspirate treated OA-chondrocytes. Senescent chondrocytes are known contributors to pathophysiological changes in OA. They are mainly located close to osteoarthritic lesions and are not found in healthy cartilage tissue (Price et al., 2002). In the senescent state they display a senescence-associated secretory phenotype (SASP), secreting high levels of pro-inflammatory cytokines, and growth factors that trigger destructive processes. In an *in vivo* study, the selective removal of p16INK4a-positive senescent cells led to a reduction in inflammation, attributed to decreased levels of SASP, indicating that reducing senescent cell populations may confer protective effects against OA-related damage (Jeon et al., 2017).

Cytokine concentration analyses in OA-patients have mainly targeted synovial fluid and, to a lesser extent, serum, revealing elevated levels of both pro-inflammatory and anti-inflammatory cytokines, chemokines, and growth factors compared to healthy individuals in synovial fluid. Additionally, correlations have been found between these elevated factors and knee pain, as well as functional limitations (Nees et al., 2019). To our knowledge, there are no existing data on cytokine concentrations in the nanofat batches used for intra-articular treatment of OA-patients. The therapeutic effects of nanofat therapy in OA-patients, whether prepared enzymatically or mechanically, appear promising. In this study, we used the sSVF of nanofat, which contains both–intact adipocytes and the secretome of processed adipocytes. Other recent publications demonstrate similar promising results using various forms of adipose-derived treatments. Ge et al. investigated a nanofat lysate, created through repeated freeze-thawing of nanofat, which improved cartilage degeneration and chondrocyte function in an experimental murine OA-model (Ge et al., 2023). Boxtel et al. demonstrated that tissue-like SVF (cells and matrix) had pro-regenerative and anti-inflammatory effects on OA-chondrocytes (Vonk et al., 2022). Additionally, Kokai et al. compared various adipose preparation methods and found that processed nanofat demonstrated anabolic and regenerative potential in human OA chondrocytes. They concluded that mechanically processed preparations could be more effective than isolated SVF cell preparations (Kokai et al., 2022). Several clinical studies have documented long lasting reductions in pain and symptoms, along with enhanced knee joint functionality (Lavagnolo et al., 2021). We observed a reduction of β-NGF and SCGF-β concentrations in the secretomes of nanofat-treated OA-chondrocytes, which is consistent with reduced pain sensation reported by patients after nanofat injection. One observed joint feature of arthritic diseases is growth of nociceptive nerve fibers along new blood vessels, which contribute to pain development. VEGF mediates the growth of new blood vessels and allows indirect growth of new nerve fibers into joint tissues (Walsh et al., 2010). Our data showed reduced VEGF secretion of nanofat-treated OA-synoviocytes and OA-chondrocytes, which may be beneficial in OA pathogenesis by preventing excessive sprouting of nociceptive nerve fibers. MCP-1 (CCL2) and GM-CSF, two factors associated with OA-pathogenesis (van Helvoort et al., 2020), were also downregulated. Data from MCP-1-deficient mice demonstrated fewer immune cells infiltrating joint tissues and consequently less cartilage damage. Additionally, a reduction in inflammation and tissue damage was observed in surgical induced OA-mice treated with an MCP-1 inhibitor (Raghu et al., 2017).

In context of an inflammatory environment, we observed a downregulation of IFN-γ in all three sSVF treatment groups OA-chondrocytes and synoviocytes, along with a reduction in IL-5 (chondrocytes), IL-7 and IL-15 (chondrocytes and synoviocytes). It is well-established that low-grade inflammation is critical in the pathogenesis of OA. Therefore, mitigating inflammatory processes and reducing pro-inflammatory cytokines are crucial for suppressing joint tissue damage (Gonçalves et al., 2022). IFN-γ has been demonstrated to induce the secretion of pro-inflammatory cytokines and MMPs in bovine chondrocytes. Additionally, it exerts a regulatory influence on human and murine osteoblasts (Gilbert et al., 2022). Thus, our data indicate a reduction of the inflammatory environment within the joint. Furthermore, we observed an increase in the secretion of TGF-β_1_ (chondrocytes and synoviocytes) and TGF β_3_ (synoviocytes). The combination of TGF-β_1_ and bone morphogenetic protein-2 (BMP-2) has been shown to promote chondrogenesis in synovial tissue explants from OA-patients, with subsequent cartilage formation (Hunziker et al., 2023). Similarly, TGF-β_3_ has been found to induce chondrogenesis of MSCs and facilitate cartilage formation (Bian et al., 2011). We propose that elevated TGF-β secretion may have therapeutic effects on damaged cartilage in OA-affected joints.

Regarding TIMPs, this study showed that nanofat treatment led to an increased secretion of TIMP-1 and TIMP-3 in both cell types. TIMPs are crucial in maintaining the balance between matrix degradation and repair, and their dysregulation can accelerate cartilage destruction in OA (Mukherjee and Das, 2024). Recent studies highlight the critical role of TIMP-3 in regulating tissue degradation and preventing cartilage damage in OA. TIMP-3 specifically inhibits several MMPs that contribute to ECM breakdown, thereby playing a key role in preserving joint integrity (Nakamura et al., 2020). Additionally, TIMP-1 has been associated with reducing inflammatory responses and mitigating pain in OA. By inhibiting MMP activity and modulating inflammatory pathways, TIMP-1 may help to alleviate the pain and inflammation that are hallmarks of OA (Knight et al., 2019). The clinical significance of this finding lies in its potential to alter the course of OA by providing symptomatic pain relief and offering regenerative effects by slowing down cartilage degradation.

However, pro-inflammatory interleukins (IL-6, IL-8), chemokines (RANTES/CCL5; Gro-α/CXCL1) and complement components (C1q, C4, C3, CFB and FH) were found to be elevated after lipoaspirate-treatment of OA-chondrocytes and OA-synoviocytes. Recent studies have demonstrated elevated levels of these molecules in the synovial fluid of OA-patients. These factors not only exacerbate inflammation but also initiate molecular changes that lead to structural alterations, like cartilage degradation, osteophyte formation, subchondral bone sclerosis and synovial inflammation, contributing to the pathogenesis of OA (Molnar et al., 2021; Dhilip and Parameswari, 2024; Holers et al., 2023; Schäfer and Grässel, 2022b; Hou et al., 2020). By elevating complement components such as C3 and CFB, along with the inhibitory factor FH, nanofat may contribute to a more balanced immune response, which could play a role in mitigating inflammation and tissue damage in OA-affected joints.

Clinically, it is recognized that knee swelling following intra-articular (i.a.) interventions usually subsides within a few days without requiring intervention. Similar observations have been made by other groups. One study reported swelling of the knee in 7% of the patients occurring less than 1 week (Panchal et al., 2018; Garza et al., 2020). However, the i. a. transplantation procedures of SVF and micro-fractured adipose tissue outlined in these studies involved administering lower volumes. Given the constrained space within the knee cavity and the clinical injection of a substantial volume (approximately 100 mL) of LC-filtered nanofat, cellular stress is expected. This leads to a transient increase in inflammatory responses. We analyzed OA-chondrocytes and OA-synoviocytes after only 24 h and 48 h of treatment. The observed elevation in inflammatory cytokines could be a natural response to the treatment, likely to resolve over time.

## Conclusion

This study demonstrated that nanofat treatment influenced the metabolic activity of chondrocytes and synoviocytes, as well as the secretion of pain- and inflammation-related factors. However, there were no significant differences in the effects between the Adinizer^®^ and Lipocube™ Nano filter systems. Based on these *in vitro* findings, both filtration systems appear suitable for clinical applications as joint treatment in OA. Further research is needed to validate these results in (pre-) clinical settings and to ensure their safety and efficacy in actual patient treatments.

Our findings indicate that nanofat may serve as a complementary or even alternative treatment to traditional anti-inflammatory therapies with NSAIDs and corticosteroids. Given the potential for fewer systemic side effects, nanofat injections could be particularly valuable for patients who are either unresponsive to conventional treatments or at risk for adverse effects from long-term medication use. Further clinical trials are essential to establish the role of nanofat in the therapeutic landscape of OA, particularly its long-term efficacy and safety profile.

### Limitations of the study

The primary limitation of this study arises from using OA-chondrocytes, OA-synoviocytes, and OA-nanofat from different donors, due to disparate medical procedures. Specifically, patients undergoing total knee replacement surgery do not receive autologous nanofat, and nanofat therapy does not necessitate knee replacement within at least 1 year after application. This can lead to a higher variability in *vitro* experiments due to the different patients' medical backgrounds. Additionally, the composition of nanofat presents a potential challenge for *in vitro* studies. While it includes anabolic physiological molecules, it may also contain environmental toxins or medication residues originating from the donor’s adipose tissue. These contaminants can influence cellular metabolism, making it difficult to reproduce experimental results consistently as each combination of cells and nanofat interacts slightly different. However, these limitations do not apply in clinical settings, as the therapy is strict autologous. Also, no non-OA sSVF could be analyzed. As a result, it was not possible to compare the concentrations of factors contained in the sSVF of OA-versus non-OA donors.

## Data Availability

The original contributions presented in the study are included in the article/Supplementary Material, further inquiries can be directed to the corresponding author.
