# Supplementary material for: Filtered and unfiltered lipoaspirates reveal novel molecular insights and therapeutic potential for osteoarthritis treatment: a preclinical in vitro study
Source: Front Cell Dev Biol. 2025 Feb 27;13:1534281. doi: 10.3389/fcell.2025.1534281 (PMC11903472; doi:10.3389/fcell.2025.1534281)
Supplement: Supplementary file 1 [file Table1.docx]

**SUPPLEMENT**

**Filtered and Unfiltered Lipoaspirates Reveal Novel Molecular Insights**

**and Therapeutic Potential for Osteoarthritis Treatment**

Alissa Behn^1^, Saskia Brendle^2, 3^, Marianne Ehrnsperger^4^, Magdalena Zborilova^4^, Thomas M. Grupp^2, 3^, Joachim Grifka^4, 5^, Nicole Schäfer^1, *^, and Susanne Grässel^1, 4, *^

^1^ Department of Orthopaedic Surgery, Experimental Orthopaedics, Centre for Medical Biotechnology (ZMB/Biopark 1), University of Regensburg, Germany

^2^ Research & Development, Aesculap AG, Tuttlingen, Germany

^3^ Department of Orthopaedic and Trauma Surgery, Musculoskeletal University Center Munich (MUM), Campus Grosshadern, LMU Munich, Munich, Germany

^4^ Department of Orthopedic Surgery, University of Regensburg, Asklepios, Germany

^5^ Director for Orthopedics and Ergonomics, Ostbayerische Technische Hochschule, Regensburg, Germany

^*^Contributed equally

**Suppl. table 1: OA-patient details of samples used for cell isolation or lipoaspirate collection.**

|  | **Chondrocyte isolation** | **Synoviocyte isolation** | **Lipoaspirate collection** |
| --- | --- | --- | --- |
| **Total** | 12 | 10 | 13 |
| **mean age** | 70 | 74  (9/10 patients) | 57 |
| **age range** | 61 - 86 | 55 – 86 | 30 - 71 |
| **male** | 8 (67 %) | 6 (60 %) | 4 (31 %) |
| **female** | 4 (33 %) | 3 (30 %) | 9 (69 %) |
| **mean BMI [m^2^/kg]** | 29.1 | 29.2 | 26.4 |
| **CRP [mg/l] mean** | 2.6 ±SD 1.8 | 2.3 ±SD 1.6 | 2.3 ±SD 1.3 |
| **Diabetes** | 0 | 0 | 0 |

**Suppl. table 2: List of used Luminex multiplex assays and ELISA-Kits.**

| **Kit name** | **Analytes** | **Company**  **# Catalogue number** |
| --- | --- | --- |
| Bio-Plex Pro Human Cytokine Screening Panel, 48-Plex | FGF basic, Eotaxin, G-CSF, GM-CSF, IFN-γ, IL-1β, IL-1ra, IL-1α, IL-2Rα, IL-3, IL-12 (p40), IL-16, IL-2, IL-4, IL-5, IL-6, IL-7, IL-8, IL-9, GRO-α, HGF, IFN-α2, LIF, MCP-3, IL-10, IL-12 (p70), IL-13, IL-15, IL-17A, IP-10, MCP-1 (MCAF), MIG, β-NGF, SCF, SCGF-β, SDF-1α, MIP-1α, MIP-1β, PDGF-BB, RANTES, TNF-α, VEGF, CTACK, MIF, TRAIL, IL-18, M-CSF, TNF-β | Bio-Rad Laboratories  #12007283 |
| Human ProcartaPlex Mix&Match 6-plex | MMP-1, MMP-13, MMP‑2, MMP-3, MMP-7, MMP-9 | Thermo Fisher Scientific  #PPX-06-MX9HKED |
| Bio-Plex Pro TGF-β 3-plex Assay | TGF-β_1_, TGF-β_2_, TGF-β_3_, | Bio-Rad Laboratories  #171W4001M |
| MILLIPLEX MAP Human TIMP Magnetic Bead Panel 2 - Immunology Multiplex Assay | TIMP-1, TIMP-2, TIMP-3 | Merck  #HTMP2MAG-54K |
| MILLIPLEX MAP Human Neuropeptide Magnetic Bead Panel - Neuroscience Multiplex Assay | α-MSH, Substance P | Merck  #HNPMAG-35K |
| Human CGRP-I EIA Kit | CGRP | RayBiotech  # EIA-CGRP-1 |
| MILLIPLEX Human Complement Panel 2 - Immunology Multiplex Assay | Complement C1q, C3, C3b/iC3b, C4, CFB, FH | Merck  # HCMP2MAG-19K |

**Suppl. table 3: Lipoaspirate concentrations of cytokines, chemokines, growth factors (Luminex 48-plex).**

| **pg/ml** | **NF** | | **AD** | | **LC** | |
| --- | --- | --- | --- | --- | --- | --- |
|  | **mean** | **± SD** | **mean** | **± SD** | **mean** | **± SD** |
| **β-NGF** | 95 | 26 | 99 | 15 | 100 | 17 |
| **CTACK** | 1.546 | 2.176 | 1.211 | 1.495 | 940 | 291 |
| **Eotaxin** | 39 | 25 | 34 | 21 | 34 | 10 |
| **FGF basic** | 1.578 | 936 | 2.417 | 1.480 | 3.472 | 2.809 |
| **G-CSF** | 1.112 | 353 | 1.429 | 520 | 1.164 | 341 |
| **GM-CSF** | 745 | 527 | 650 | 459 | 777 | 1.085 |
| **GRO-α** | 10.424 | 5.732 | 11.434 | 3.916 | 12.767 | 4.534 |
| **HGF** | 6.869 | 4.331 | 5.932 | 3.660 | 7.472 | 3.921 |
| **IFN-α2** | 215 | 20 | 195 | 80 | 221 | 23 |
| **IFN-γ** | 225 | 46 | 223 | 102 | 244 | 99 |
| **IL-10** | 116 | 18 | 121 | 8 | 138 | 56 |
| **IL-12(p40)** | 1.717 | 267 | 1.766 | 127 | 1.856 | 228 |
| **IL-12(p70)** | 171 | 21 | 179 | 10 | 192 | 24 |
| **IL-13** | 79 | 33 | 77 | 31 | 85 | 36 |
| **IL-15** | 3.877 | 1.154 | 4.097 | 1.225 | 3.574 | 1.431 |
| **IL-16** | 6.489 | 3.241 | 8.384 | 5.817 | 8.426 | 4.993 |
| **IL-18** | 287 | 186 | 457 | 396 | 691 | 619 |
| **IL-1ra** | 9.604 | 5.880 | 13.382 | 13.654 | 16.099 | 10.910 |
| **IL-1α** | 539 | 80 | 553 | 50 | 580 | 46 |
| **IL-1β** | 44 | 19 | 48 | 18 | 49 | 21 |
| **IL-2Ra** | 276 | 121 | 277 | 101 | 296 | 103 |
| **IL-3** | 12 | 3 | 12 | 2 | 12 | 1 |
| **IL-4** | 30 | 10 | 30 | 9 | 31 | 11 |
| **IL-7** | 438 | 99 | 454 | 54 | 518 | 143 |
| **IL-9** | 972 | 744 | 1.185 | 522 | 1.546 | 617 |
| **IP-10** | 1.475 | 2.333 | 879 | 1.160 | 551 | 103 |
| **LIF** | 655 | 222 | 776 | 90 | 683 | 282 |
| **MCP-1** | 362 | 207 | 329 | 174 | 379 | 174 |
| **MCP-3** | 58 | 11 | 56 | 7 | 60 | 11 |
| **M-CSF** | 393 | 218 | 443 | 184 | 491 | 267 |
| **MIF** | 82.337 | 30.309 | 88.583 | 33.824 | 88.337 | 27.975 |
| **MIG** | 6.159 | 7.973 | 6.074 | 5.729 | 6.067 | 6.580 |
| **MIP-1α** | 7 | 4 | 13 | 9 | 9 | 4 |
| **MIP-1β** | 691 | 702 | 860 | 587 | 1.113 | 600 |
| **PDGF-bb** | 2.574 | 2.252 | 1.935 | 1.027 | 3.023 | 2.901 |
| **RANTES** | 11.802 | 18.316 | 9.399 | 7.466 | 23.055 | 16.885 |
| **SCF** | 690 | 336 | 594 | 277 | 654 | 325 |
| **SCGF-β** | 61.080 | 36.035 | 62.329 | 35.347 | 70.750 | 32.155 |
| **SDF-1α** | 2.108 | 1.740 | 1.852 | 683 | 1.882 | 451 |
| **TNF-α** | 667 | 313 | 657 | 247 | 719 | 342 |
| **TNF-β** | 1.245 | 1.354 | 1.455 | 888 | 1.858 | 908 |
| **TRAIL** | 801 | 607 | 773 | 360 | 1.051 | 678 |
| **VEGF** | 2.717 | 1.206 | 2.779 | 709 | 2.671 | 982 |

**Suppl. table 4: Lipoaspirate concentrations of MMPs, TIMPs, TGF-β, sensory neuropeptides, complement components (pg/ml).**

| **pg/ml** | **NF** | | | **AD** | | | **LC** | |
| --- | --- | --- | --- | --- | --- | --- | --- | --- |
|  | **mean** | **± SD** | | **mean** | **± SD** | | **mean** | **± SD** |
| **TIMP-1** | 12.246 | 4.615 | | 11.581 | 3.926 | | 12.708 | 4.917 |
| **TIMP-2** | 30.202 | 5.490 | | 24.683 | 9.724 | | 34.962 | 5.081 |
| **TIMP-3** | 7.169 | 2.492 | | 6.648 | 3.330 | | 5.922 | 2.568 |
|  |  |  | |  |  | |  |  |
| **MMP-1** | 191 | 302 | | 94 | 92 | | 191 | 178 |
| **MMP-2** | 249 | 93 | | 254 | 75 | | 352 | 193 |
| **MMP-3** | 188 | 117 | | 240 | 156 | | 244 | 97 |
| **MMP-7** | 88 | 89 | | 61 | 47 | | 89 | 45 |
| **MMP-9** | 130 | 45 | | 113 | 40 | | 95 | 29 |
|  |  |  | |  |  | |  |  |
| **TGF-β_1_** | 527 | 354 | | 604 | 88 | | 437 | 147 |
| **TGF-β_2_** | 38 | 8 | | 41 | 2 | | 39 | 5 |
| **TGF-β_3_** | 8 | 3 | | 9 | 1 | | 6 | 2 |
|  |  |  | |  |  | |  |  |
| **CGRP** | 146 | 95 | | 114 | 61 | | 147 | 61 |
| **SP** | 139 | 92 | | 343 | 504 | | 253 | 165 |
|  |  |  | |  |  | |  |  |
| **C1q** | 552 | 61 | | 528 | 116 | | 579 | 88 |
| **C3** | 77 | 103 | | 42 | 29 | | 8 | 5 |
| **C3b/iC3b** | 1.891 | 1.450 | | 1.051 | 979 | | 1.226 | 731 |
| **C4** | 811 | 47 | | 854 | 97 | | 846 | 74 |
| **CFB** | 555 | 176 | | 571 | 110 | | 487 | 88 |
| **CFH** | 981 | 129 | | 970 | 107 | | 885 | 178 |
| **TIMP-2:** AD vs LC *p< 0.05 | | | **TGF-β_3_:** AD vs LC *p< 0.05 | | | **C3:** NF/AD vs LC *p< 0.05 | | |
